# Functional Analysis of Ectodysplasin-A Mutations in X-Linked Nonsyndromic Hypodontia and Possible Involvement of X-Chromosome Inactivation

**DOI:** 10.1155/2021/7653013

**Published:** 2021-09-09

**Authors:** Yuhua Pan, Ting Lu, Ling Peng, Qi Zeng, Xiangyu Huang, Xinchen Yao, Buling Wu, Fu Xiong

**Affiliations:** ^1^Department of Stomatology, Nanfang Hospital, Southern Medical University, Guangzhou 510515, China; ^2^Department of Pediatric Dentistry, Guanghua School of Stomatology, Guangdong Provincial Key Laboratory of Stomatology, Sun Yat-sen University, Guangzhou, China; ^3^Shenzhen Stomatology Hospital (Pingshan), Southern Medical University, 143 Dongzong Road, Pingshan District, Shenzhen 518118, China; ^4^Department of Medical Genetics, School of Basic Medical Sciences, Southern Medical University, Guangzhou 510515, China; ^5^Guangdong Provincial Key Laboratory of Single Cell Technology and Application, Guangzhou, Guangdong, China; ^6^Department of Fetal Medicine and Prenatal Diagnosis, Zhujiang Hospital, Southern Medical University, Guangzhou, China; ^7^Experimental Education/Administration Center, School of Basic Medical Science, Southern Medical University, Guangzhou 510515, China

## Abstract

**Background:**

Mutations of the Ectodysplasin-A (EDA) gene are generally associated with syndrome hypohidrotic ectodermal dysplasia or nonsyndromic tooth agenesis. The influence of EDA mutations on dentinogenesis and odontoblast differentiation has not been reported. The aim of this study was to identify genetic clues for the causes of familial nonsyndromic oligodontia and explore the underlying mechanisms involved, while focusing on the role of human dental pulp stem cells (hDPSCs).

**Materials and Methods:**

Candidate gene sequences were obtained by PCR amplification and Sanger sequencing. Functional analysis was conducted, and the pathogenesis associated with *EDA* mutations in hDPSCs was investigated to explore the impact of the identified mutation on the phenotype. Capillary electrophoresis (CE) was used to detect X-chromosome inactivation (XCI) in the blood of female carriers.

**Results:**

In this study, we identified an *EDA* mutation in a Chinese family: the missense mutation c.1013C>T (Thr338Met). Transfection of hDPSCs with a mutant *EDA* lentivirus decreased the expression of EDA and dentin sialophosphoprotein (DSPP) compared with transfection of control EDA lentivirus. Mechanistically, mutant EDA inhibited the activation of the NF-*κ*B pathway. The CE results showed that symptomatic female carriers had a skewed XCI with a preferential inactivation of the X chromosome that carried the normal allele.

**Conclusions:**

In summary, we demonstrated that *EDA* mutations result in nonsyndromic tooth agenesis in heterozygous females and that, mechanistically, EDA regulates odontogenesis through the NF-*κ*B signalling pathway in hDPSCs. Due to the large heterogeneity of tooth agenesis, this study provided a genetic basis for individuals who exhibit similar clinical phenotypes.

## 1. Introduction

Tooth agenesis, the congenital absence of one or more permanent teeth, is the most common abnormality affecting the function and aesthetics of patients. Congenital tooth agenesis has been divided into nonsyndromic hypodontia (NSH) and syndromic hypodontia (SH) based on the systemic conditions of the patient [1, 2]. Patients with NSH only exhibit tooth-related symptoms (including tooth number abnormalities and tooth morphogenesis abnormalities). Based on the number of missing teeth (not including third molars), tooth agenesis can be classified as hypodontia (less than 6 missing teeth), oligodontia (6 or more missing teeth), and anodontia (all teeth missing) [3, 4]. To date, NSH has been reported to be associated with mutations in ectodysplasin-A (*EDA*), ectodysplasin-A receptor (*EDAR*), EDAR-associated death domain (*EDARADD*), wnt family member 10A (*WNT10A*), wnt family member 10B (*WNT10B*), paired box 9 (*PAX9*), msh homeobox 1 (*MSX1*), axis inhibition protein 2 (*AXIN2*), and inhibitor of nuclear factor kappa B kinase regulatory subunit gamma (*IKBKG*) [5–7]. Among these, *EDA* mutations could cause SH, which appears as an X-linked hypohidrotic ectodermal dysplasia (XLHED) clinical feature, and they have also been linked to isolated tooth agenesis, most likely due to complete or partial disruption of the EDA signalling pathway [8, 9].

The *EDA* gene is located on chromosome Xq12-13.1 and encodes a protein that belongs to the tumour necrosis factor superfamily of ligands [10]. It contains 12 exons, and 8 of these exons encode the two main proteins: EDA1, which binds to the EDA receptor (EDAR), and EDA2, a variant that is two amino acids shorter and binds exclusively to a receptor called XEDAR [11]. In addition, both EDAR and XEDAR receptors activate the NF-*κ*B pathway. The EDA/EDAR/NF-*κ*B pathway has previously been found to be required for normal embryogenesis, particularly in tooth growth and morphogenesis at different stages of tooth development [12]. Indeed, previous investigations on the impact of syndrome-causing *EDA* mutations have shown that most syndrome-causing EDA mutations are predicted to ultimately cause receptor signalling to be eliminated. However, some observations of an *EDA* mutation in a family with X-linked, recessive, nonsyndromic tooth agenesis seem to indicate that the expression, receptor binding, and signalling capability of the mutant EDA1 proteins were only impaired rather than abolished [13–15].

In our studies, we found that a known *EDA* mutation c.1013C>T (p.T338M) was associated with nonsyndromic tooth agenesis, and the mutation was located in the TNF homology domain, which was first reported in 2008 [16]. However, compared with the previously reported case, the cases herein exhibited differences in the clinical phenotype compared. In our studies, the heterozygous female carriers were missing only one tooth rather than eight teeth. Notably, in previous studies, heterozygous female carriers with *EDA* mutations have usually presented a highly variable and milder dental phenotype compared with those in our studies [16, 17]. Some researchers have suggested that in heterozygous female carriers with *EDA* mutations, dental phenotype variability may occur due to the X-chromosome inactivation (XCI) differential pattern, which needs further exploration [18].

XCI is a random process by which one of the two copies of the female mammal X chromosome is inactivated [19]. Distortion of the X inactivation occurs by chance, as a result of this initial inactivation process; by selection, as a result of cellular survival or proliferation advantage; or by mutation of the X inactivation apparatus [20]. XCI affects the maternal or paternal X chromosome and follows a ratio of approximately 50 : 50 in normal females (random XCI) [21]. A skewed XCI can cause female carriers of an X-linked recessive disease to have different levels of protein expression, which is derived from the mutated allele, and experience partial or complete symptoms of the disorder [22–24]. However, detailed descriptions of X-linked tooth agenesis phenotypes and the skewing of X inactivation have been minimal.

Since they were first defined, human dental pulp stem cells (hDPSCs) have been found to be essential for dentin formation and odontogenesis, as they have a high proliferative potential and the capacity for self-renewal and experience multilineage differentiation [25]. Therefore, hDPSCs represent a valuable model for the investigation of odontoblastic differentiation and the mechanisms by which EDA affects the function of such cells. In addition, the EDA mutation site has been reported before but the function of EDA in hDPSCs and whether the mutant EDA affects the function of hDPSCs have not been determined.

In the present study, we identified a known missense mutation of *EDA* in a Chinese family, which manifested as X-linked, recessive NSH, and explored how this EDA mutation affected the function of hDPSCs. In addition, we investigated genotypes and phenotypes in patients with X-linked NSH, and we assessed a possible relationship between the severity of clinical symptoms and XCI in female carriers.

## 2. Materials and Methods

### 2.1. Pedigree and Clinical Diagnosis

Two Chinese patients with oligodontia, aged 10 and 11, were identified in the Department of Endodontics of Nanfang Hospital (Guangdong, China). Panoramic radiographs confirmed the diagnosis of nonsyndromic hypodontia. A pedigree construction was created by conducting clinical examinations of the available family members and by conducting interviews. A total of 6 family members participated in this study. Tooth agenesis could be traced back three generations. All subjects gave informed consent, and the study was approved by the Ethics Committee of Southern Medical University (No. NFEC-2021-137).

### 2.2. Identification of Mutations

To identify disease-associated mutations, we used a standard phenol/chloroform extraction method to extract genomic DNA from the peripheral blood of the proband individual and his family members. Screening of pathogenic mutations was performed using polymerase chain reaction (PCR) amplification and by sequencing the complete exons and exon–intron boundaries of EDA. PCR products were submitted to Invitrogen (Shanghai, China) for sequencing. Then, to confirm the causative mutation, cosegregation analyses were performed for all family members.

### 2.3. Construction of EDA Expression Vectors, Site-Directed Mutagenesis, and Vector Lentivirus Package

Full-length human EDA was cloned into the expression vector pcDNA3.1 (Invitrogen), and EDA-wild type (WT) was obtained. The c.1013C>T mutant (MUT) was generated using the following primers: forward, 5′-ACACGCAGCATCGAGATGGGCAAGACCAACTAC-3′; and reverse, 5′-GTAGTTGGTCTTGCCCATCTCGATGCTGCGTGT-3′, which replaced threonine (Thr) with methionine (Met). Then, the plasmid vectors were packaged into lentiviruses and the control lentivirus (Ctrl), which were synthesized by GeneChem (Shanghai, China), to establish a stably transfected cell line in the subsequent studies.

### 2.4. Cell Culture and Odontoblastic Differentiation

Isolation of hDPSCs was performed as described elsewhere [26]. For the odontoblastic differentiation experiments, the cells were cultured in odontogenic medium (OM), consisting of Dulbecco's modified Eagle's medium (DMEM, Gibco, Invitrogen, NY, USA), 10% fetal bovine serum (FBS, Gibco), 50 mg/ml ascorbic acid (Sigma-Aldrich, St. Louis, MO, USA), 5 mM b-glycerophosphate (Sigma-Aldrich), and 10 nM dexamethasone (Sigma-Aldrich).

### 2.5. Cell Infection

We overexpressed WT and MUT Flag-EDA samples in hDPSCs with lentivirus following the manufacturer's instructions. hDPSCs were seeded in 12-well plates at a density of 10^5^ cells/well and grown for 24 h. The cells were infected with a multiplicity of infection (MOI) of 20 in the presence of 5 mg/ml polybrene for 10 h at 37°C and 5% CO_2_.

### 2.6. Western Blot Analysis

Cells were harvested and lysed in RIPA buffer (Beyotime, Nanjing, China) supplemented with protease inhibitors (Beyotime). Total protein (20 *μ*g) was separated on a 10% SDS-polyacrylamide gel and transferred onto a polyvinylidene difluoride (PVDF) membrane (Amersham, Little Chalfont, UK). The membranes were blocked for 1 h with 5% skim milk and incubated overnight at 4°C with anti-FLAG, anti-GAPDH (1 : 1000; Proteintech, China), anti-DSPP (1 : 1000; Santa Cruz Biotechnology), anti-p65 (1 : 1000; SAB, China), anti-p-p65 (1 : 1000; SAB, China), anti-IkBa (1 : 1000; SAB, China), and anti-p-IkBa (1 : 1000; SAB, China) antibodies. The next day, the membranes were incubated for 1 h at 37°C with the corresponding secondary antibodies (Proteintech, China), and the immunoreactive proteins were visualized with an ECL Kit (Beyotime Biotech, Shanghai, China) according to the manufacturer's instructions.

### 2.7. Quantitative Real-Time PCR (RT-qPCR)

Quantitative real-time PCR (RT-qPCR) was applied to examine the expression of *EDA* and dentin sialophosphoprotein (*DSPP*). Total RNA was reverse-transcribed using the PrimeScript First Strand cDNA Synthesis Kit (TaKaRa Biotechnology, China), and RT-qPCR was carried out with SYBR Premix DimerEraser (TaKaRa Biotechnology, China) on a LightCycler 480 (Roche, Indianapolis, USA). The DSPP primers were as follows: forward primer, 5′-GGCCATTCCAGTTCCTCAAAG-3′; and reverse primer, 5′-TGCACCAGGACACCACTTTC-3′. The EDA primers were as follows: forward primer, 5′-CTCGAGAAAACCAGCCAGC-3′; and reverse primer, 5′-CAGTCATTGAGCACTCCACC-3′. The GAPDH primers were as follows: forward: 5′-GTGAAGGTCGGAGTCAACG-3′ and reverse: 5′-TGAGGTCAATGAAGGGGTC-3′. Details of the PCR conditions are indicated as follows. In brief, a 20 *μ*l reaction mixture included 2 *μ*l cDNA, 10 *μ*l SYBR Premix DimerEraser, 0.8 *μ*l 2× primer pairs (20 *μ*M), and 7.2 *μ*l ddH_2_O. The reaction conditions are as follows: a 30 s initial denaturation at 95°C was followed by 40 cycles consisting of 10 s at 95°C, 30 s at 60°C, and a final elongation at 72°C for 5 min, ending with a holding period at 16°C. Gene expression levels were calculated using the 2^-*ΔΔ*CT^ method. To confirm the reproducibility of the results, transfection and real-time PCR assays were repeated three times.

### 2.8. Alizarin Red S (ARS) Staining

The number of calcium nodules formed by hDPSCs after transfection with EDA lentivirus vector, WT, or MUT EDA was analysed by Alizarin Red S (ARS) staining. After being cultured in OM for 14 days, the cells were fixed using 60% isopropanol (Macklin, China) for 30 min and stained with 1% ARS (Sigma-Aldrich, USA) at room temperature. After washing several times with deionized water, calcium nodules were observed with a microscope (Crystal violet, Amresco, Solon, OH).

### 2.9. Detecting Skewing of X Inactivation

The XCI pattern was analysed using the human androgen receptor (HUMARA) assay [27], which utilizes the highly polymorphic CAG repeat sequence in the first exon of the androgen receptor (AR) gene. With digestion of the methylation-sensitive restriction enzyme HpaII and differential PCR amplification, the AR gene was used as a marker of skewed XCI. In brief, DNA samples were obtained from the peripheral venous blood of the female carrier (II3) and her affected father (I3). For each sample, 600 ng of DNA was digested with HpaII. Digested products, together with nondigested DNA, were used as templates for the amplification of the AR polymorphic repeat sequence using the following fluorescence primers: *AR* forward, 5′-GCTGTGAAGGTTGCTGTTCCTCAT-3′; and *AR* reverse, 5′-TCCAGAATCTGTTCCAGAGCGTGC-3′. We identified the digestion efficiencies with *MIC2* as an internal reference. The amplification products of *MIC2* could be observed when the DNA was not completely digested. The primers were as follows: *MIC2F* forward, 5′-AGAGGTGCGTCCGATTTTTCCC-3′; and *MIC2F* reverse, 5′-ACCGCCGCAGATGGACAATT-3′. The products were analysed by capillary electrophoresis (Beckman Coulter, USA).

### 2.10. Bioinformatics

To further confirm the mutant EDA function, 3D structures of the WT and mutant EDA were predicted in silico using I-TASSER (http://zhanglab.ccmb.med.umich.edu/I-TASSER/), and the functional effects of the mutant protein were estimated with PolyPhen-2 (http://genetics.bwh.harvard.edu/pph2/).

### 2.11. Statistical Analysis

Statistical analyses were performed using GraphPad Prism software (GraphPad Software Inc., San Diego, CA, USA). The statistical significance between the two groups was determined using an independent samples *t*-test, and significance between more than two groups was analysed by one-way ANOVA. *P* < 0.05 was considered significant.

## 3. Results

### 3.1. Clinical Phenotypes

**Figure 1:**
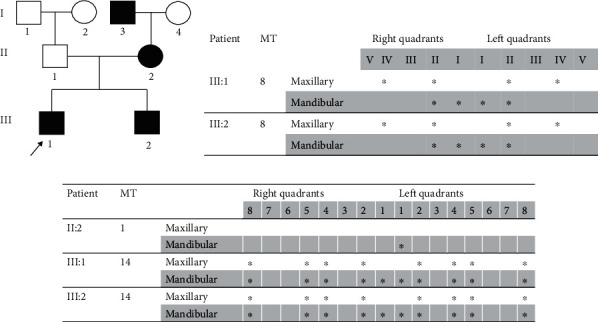
(a) Pedigree of the family. Males are shown as squares, and females are shown as circles. An arrow indicates the proband individual, and the black symbols indicate the affected individuals. (b) Summary of the missing primary teeth of the two male patients who had panoramic radiographs. (c) Summary of the missing permanent teeth of the three patients who had panoramic radiographs. MT: missing teeth.

**Figure 2:**
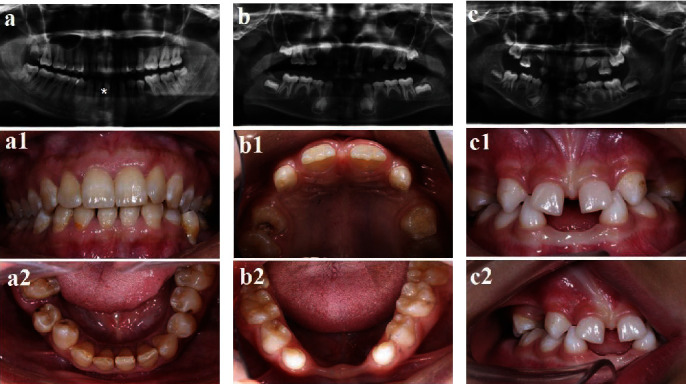
Dental phenotype and panoramic radiographs of the patients. (a–c) Panoramic radiographs of II:2, III:1, and III:2. (a1, a2; b1, b2; c1, c2) Intraoral photos of II:2, III:1, and III:2.

Clinical and radiological examinations revealed that the proband individual presented with 8 deciduous teeth missing and 18 permanent teeth missing (Figures 1(b) and 1(c) and Figures 2(b1) and 2(b2)). His brother (Figures 1(b) and 1(c) and Figures 2(c1) and 2(c2)) had the same phenotypes as he did, but his mother (Figures 1(b) and 1(c) and Figures 2(a1) and 2(a2) had only one missing tooth. The proband's father and grandmother had no missing teeth. For the grandfather, we only collected blood and did not perform a radiological examination because of his old age. According to a description from other family members, the grandfather had similar oral manifestations. All family members denied having any medical history of abnormalities in other organs including the sweat glands, hair, skin, and nails or any other systemic disorder, indicating an NSH phenotype.

### 3.2. Mutation Analysis

**Figure 3:**
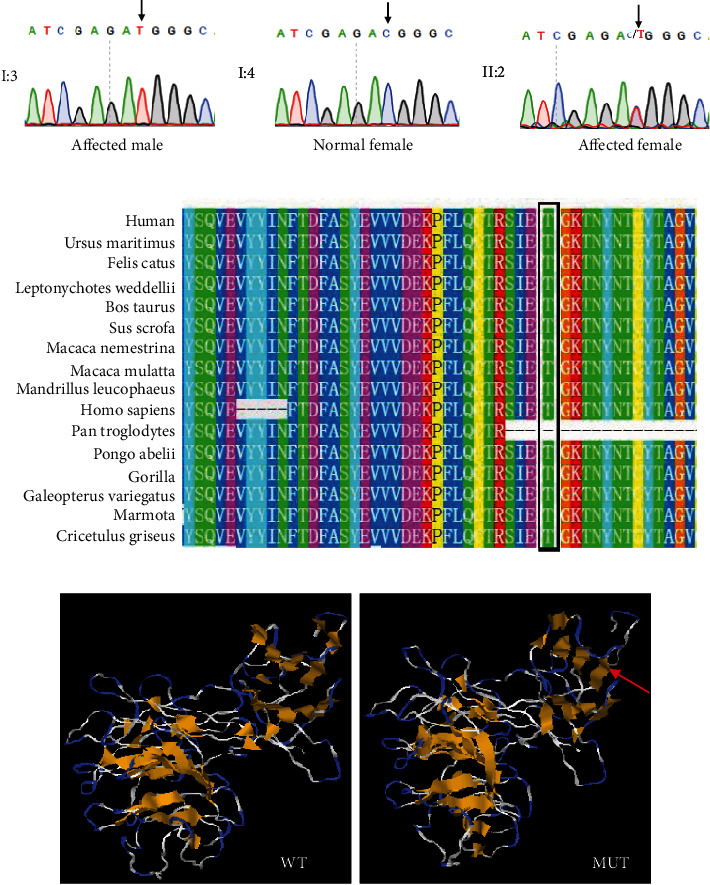
Mutation analysis. (a) Mutation screening. Sanger sequencing results of wild-type and mutant EDA. (b) Conservation of the mutant EDA across species. (c) Three-dimensional models of wild-type and mutant EDA.

In this family, Sanger sequencing detected no mutations in *PAX9*, *AXIN2*, *MSX1*, or *WNT10A*, which were previously reported as gene candidates for isolated tooth agenesis. However, we found the reported variant c.1013C > T (p. Thr338Met) in exon 8 of *EDA*. Sequencing analyses of the Thr338Met mutation were performed on all participants (Figure 3(a)). The affected female patient (II2) was a hemizygous carrier for p. Thr338Met, and all affected male individuals (III1, III2, and I3) were determined to be homozygous mutants, while the unaffected grandmother (I4) did not carry this mutation (Figures 1 and 3(a)).

The Thr338 position is highly conserved in the other known EDA proteins (Figure 3(b)), suggesting that it has an important function in the protein. I-TASSER analyses showed that the EDA c.1013C > T mutation changed the tertiary structure of the protein, causing a conformational change of the homotrimer (Figure 3(c)).

### 3.3. Mutation of EDA Inhibited Osteo-/Odontogenic Differentiation of hDPSCs

**Figure 4:**
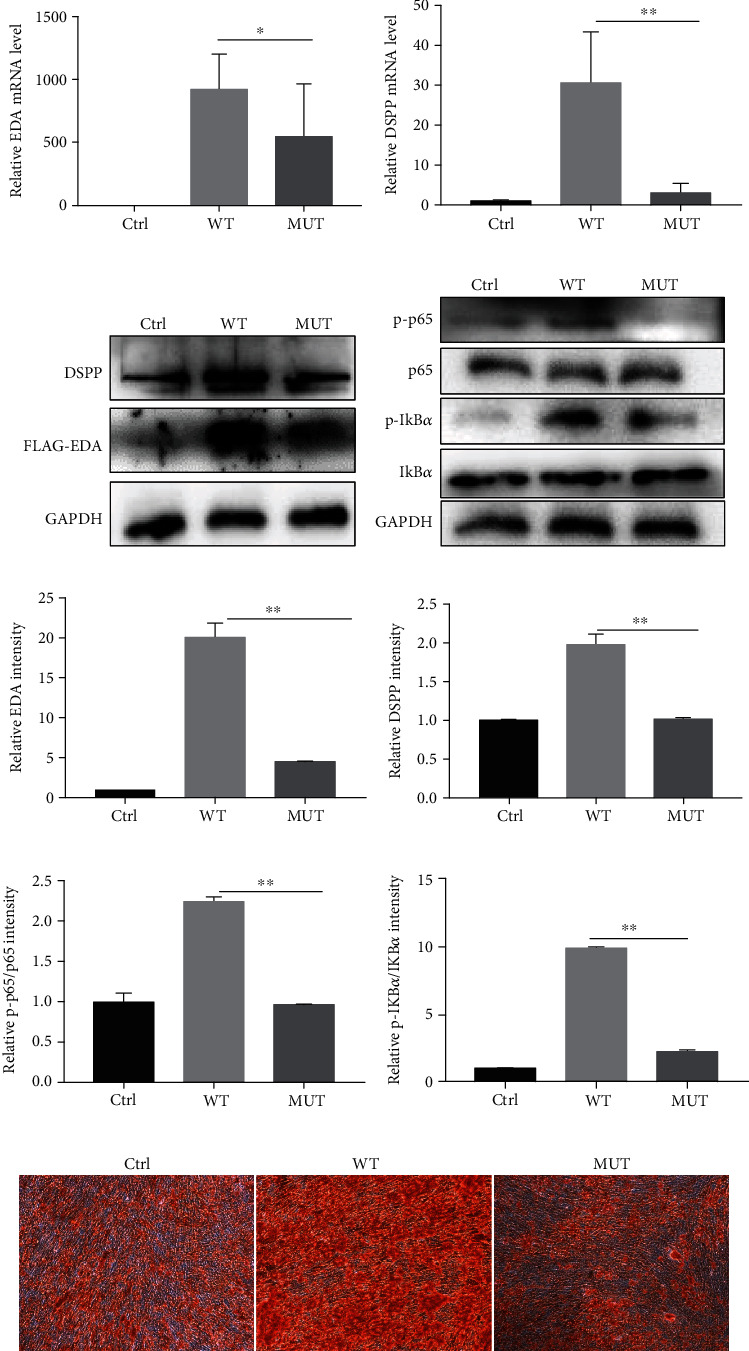
Effects of EDA on odontogenic differentiation of hDPSCs. (a, b) mRNA expression level of *EDA* and *DSPP* in hDPSCs. There were significant differences in the mRNA levels of wild-type and mutant EDA. (c) Western blot analysis of EDA and DSPP expression. (d) Human DPSCs transfected with mutant EDA expressed lower levels of p-p65 and p-IkB*α* than hDPSCs transfected with wild-type EDA. (e–h) Relative intensity of DSPP, EDA, p-p65, and p-IkB*α*. (i) Calcium nodule deposition of hDPSCs after transfection was examined by Alizarin Red S staining. Mutant EDA showed a decreased capacity of odontogenic differentiation compared with those transfected with wild-type EDA. Data are expressed as the mean ± SD. Each experiment was repeated three times with *n* ≥ 3 samples per group. ^∗^*P* < 0.05 and ^∗∗^*P* < 0.01.

To investigate whether mutant EDA affects the function of hDPSCs, hDPSCs were transfected with Ctrl, WT, and MUT and cultured in odontogenesis induction medium for 14 days, and calcium nodule deposition was then examined by ARS staining. We found that, after 14 days of odontogenic induction, EDA overexpression increased the expression of DSPP and calcium nodule deposition, while the mutant EDA showed a decreased capacity for odontogenic differentiation compared with WT EDA (Figures 4(a)–4(c) and 4(i)).

### 3.4. EDA Regulated Odontogenesis of hDPSCs through the NF-*κ*B Pathway

Western blot results showed that WT transfection significantly increased the p65 and I*κ*B*α* phosphorylation levels compared with Ctrl transfection, while mutant EDA transfection decreased the p65 and I*κ*B*α* phosphorylation levels compared with wild-type EDA transfection (Figure 4(d)). These results reveal, for the first time, that EDA regulates the odontogenesis of hDPSCs via the NF-*κ*B pathway, thus leading to the oligodontia phenotype in the patients.

### 3.5. Analysis of XCI Skewing

**Figure 5:**
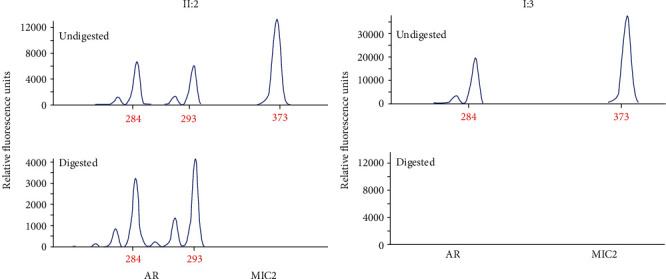
Skewing of the X inactivation analysis. The HUMARA assay was performed on genomic DNA extracted from the heterozygous female patient (II:2) and her father's (I:3) peripheral blood. The DNA was digested with and without a methylation-sensitive restriction enzyme (HpaII), amplified by PCR using FAM-labelled primers specific to the HUMARA locus, and analysed by capillary electrophoresis.

The results of capillary electrophoresis showed that there were three II2 products before DNA digestion: 284 bp (paternal source), 293 bp (maternal source), and 373 bp (internal marker MIC2). There was no MIC2 product when the DNA was completely digested. Similarly, because males have only one X chromosome that cannot be methylated, there were two products before digestion and no products after digestion (Figure 5). In summary, the results indicated that the female carrier's X chromosome obtained from the paternal donor had an inactivation rate of 39.3%.

## 4. Discussion

We identified a known missense mutation (c.1013C>T, p.T338M) of *EDA* in a Chinese family that manifested as X-linked nonsyndromic hypodontia [16]. Although previous studies have identified more than one hundred mutations related to XLHED in *EDA*, only a few *EDA* variants have been implicated in nonsyndromic tooth agenesis [28]. Patients with tooth agenesis not only have oral functional problems, including temporomandibular joint dysfunction and problems with mastication, swallowing, or speech, but can also have psychological problems due to their appearance. The most common treatments have depended on orthodontic therapy [29]. Therefore, identification of the pathogenesis and prevention and treatment have important implications for promoting the quality of life in patients with tooth agenesis.

Human DPSCs are known to play an important role in tooth homeostasis and formation. The odontogenic abilities of hDPSCs are crucial for this physiological process [30]. Recently, clinical trials for hDPSC-mediated therapies have rapidly developed, and there is a high potential for using hDPSCs as a novel therapy for many diseases, such as periodontal disease [31], neurological diseases [32], and stroke [33, 34]. However, there is no research available regarding the effect of EDA and its mutant on the odontogenic differentiation abilities of hDPSCs.

In the present study, we first confirmed intrinsic EDA expression and investigated its role in the odontogenic differentiation of hDPSCs. To further analyse how the mutation of *EDA* caused tooth agenesis, we constructed wild-type EDA expression vectors and performed site-directed mutagenesis to obtain mutant EDA expression vectors, which were packaged into lentiviruses to allow hDPSCs to be more easily infected. Compared with Ctrl transfection, wild-type EDA transfection increased calcium nodule formation and DSPP expression. Human DPSCs transfected with mutant EDA lentivirus showed a decreased capacity for odontogenic differentiation with a lower expression level of DSPP compared with those transfected with wild-type EDA lentivirus. To our knowledge, this study is the first to demonstrate the effects of EDA and its mutant on hDPSCs.

Previous studies have confirmed that the NF-*κ*B pathway is a downstream effector of EDA [9]. The EDA/NF-*κ*B pathway has been found to be important for tooth growth, and mutations in several genes in this pathway have been reported to be responsible for ectodermal dysplasia, such as syndromic tooth agenesis [35]. Therefore, dysfunctional EDA might severely impair NF-*κ*B activation in various stages during tooth development; thus, it can lead to abnormalities in the permanent dentition and even in the primary dentition. However, the question of whether EDA and mutant EDA affect the NF-*κ*B pathway of hDPSCs has yet to be elucidated. In our studies, we confirmed the relationship between the *EDA* mutation and the NF-*κ*B signalling pathway in hDPSCs. However, why the *EDA* mutation can partially, and not completely, abolish the activation of NF-*κ*B needs to be further investigated.

Previous studies on *EDA* mutations have suggested that male patients' clinical symptoms and phenotypes are more severe than those of female carriers [16–18]. Skewed inactivation of the X chromosome, with overexpression of the mutant gene in affected females, could theoretically be the cause of the oligodontia phenotype [19–21]. In this family, the female carrier (II2) had only one missing tooth, which was different from the previously reported case (several missing teeth) with the same mutation site, while her sons with the mutation had a severe phenotype. To verify whether skewed X inactivation may be involved in the pathogenesis of the disease in female carriers in this family, we conducted an XCI analysis in the peripheral blood of the female carrier. Although the results showed only a 39.3 : 61.7 skewing of X inactivation in the peripheral blood DNA as measured by androgen receptor allele methylation, we can partly explain why the female carrier has a milder phenotype. We understand that if the pattern of inheritance in our pedigree is X-linked recessive and the XCI rate is 50 : 50, the female carrier will not have tooth agenesis. If, however, there is a slightly skewed inactivation of the X chromosome, the female carrier will have mild phenotypes of tooth agenesis. Similarly, if inactivation of the X chromosome is severely skewed (≥80 : 20), the female carrier will have severe tooth agenesis phenotypes.

## 5. Conclusion

In conclusion, we verified the pathogenicity of the *EDA* c.1013C>T mutation in a three-generation Chinese family with X-linked nonsyndromic hypodontia. Subsequent in vitro studies, for the first time, verified the odontogenic function of EDA and its mutant in hDPSCs. The mild phenotype of the female might have involved XCI. Understanding the possible mechanisms will provide insights for novel therapeutic strategies for patients with tooth agenesis caused by *EDA* mutations in the future.

## Data Availability

The data that support the findings of this study are available from the corresponding author on request.

## Abbreviations

EDA: Ectodysplasin-A
hDPSCs: Human dental pulp stem cells
CE: Capillary electrophoresis
XCI: X-chromosome inactivation
DSPP: Dentin sialophosphoprotein
NSH: Nonsyndromic hypodontia
SH: Syndromic hypodontia
XLHED: X-linked hypohidrotic ectodermal dysplasia
PCR: Polymerase chain reaction
WT: Wild type
MUT: Mutant type
OM: Odontogenic medium
DMEM: Dulbecco's modified Eagle's medium
FBS: Fetal bovine serum
MOI: Multiplicity of infection
ARS: Alizarin Red S staining
HUMARA: Human androgen receptor
AR: Androgen receptor
GAPDH: Glyceraldehyde-3-phosphate dehydrogenase
PAX9: Paired box 9
WNT10A: Wnt family member 10A
MSX1: Msh homeobox 1
AXIN2: Axis inhibition protein 2
NF-*κ*B: Nuclear factor kappa-B
MIC2: Micronemal protein 2
Thr: Threonine
Met: Methionine
EDAR: Ectodysplasin-A receptor
EDARADD: EDAR-associated death domain
IKBKG: Inhibitor of nuclear factor kappa B kinase regulatory subunit gamma.
